# Effect of homocysteine on intestinal permeability in rats with experimental colitis, and its mechanism

**DOI:** 10.1093/gastro/gou022

**Published:** 2014-04-27

**Authors:** Hao Ding, Qiao Mei, Hui-Zhong Gan, Li-Yu Cao, Xiao-Chang Liu, Jian-Ming Xu

**Affiliations:** ^1^Department of Gastroenterology, The First Affiliated Hospital of Anhui Medical University and The Key Laboratory of Digestive Diseases of Anhui Province, Hefei, China ^2^Department of Pathology, The First Affiliated Hospital of Anhui Medical University, Hefei, China

**Keywords:** homocysteine, colitis, trinitrobenzene sulfonic acid, intestinal permeability

## Abstract

**Objective:** To investigate the effect of homocysteine (Hcy) on intestinal permeability in rats with TNBS/ethanol-induced colitis and elucidate its mechanism.

**Methods:** Sprague-Dawley rats were divided into four groups: normal, normal + Hcy injection, TNBS model, and TNBS model + Hcy injection. Experimental colitis was induced by trinitrobenzene sulfonic acid (TNBS) in 50% ethanol; rats were injected subcutaneously with Hcy from the first day after the induction of experimental colitis on 30 consecutive days. To determine the severity of colitis, the disease activity index (DAI) was evaluated; colon tissues were collected for the detection of the activity of myeloperoxidase (MPO) and the contents of MDA, IL-1β, IL-6, TNF-α, MMP-2, and MMP-9. Intestinal epithelial permeability was assessed with Evans blue (EB) dye. The levels of Hcy in plasma and colon mucosa were measured by high-performance liquid chromatography-fluorescence detection (HPLC-FD).

**Results:** Compared with the normal group, the DAI scoring and MPO activity, contents of MDA, IL-1β, IL-6, TNF-α, MMP-2, MMP-9 in the colon and EB in the small intestine were significantly increased in the TNBS group (P < 0.01). Compared with the TNBS model group, the DAI scoring, plasma and colonic mucosa Hcy levels, MPO activity and contents of MDA, IL-1β, IL-6, TNF-α, MMP-2, MMP-9 in colon and EB in small intestine were significantly increased in the TNBS-induced colitis rats with simultaneous Hcy injection (P < 0.01).

**Conclusion:** Hcy can increase intestinal permeability and aggravate inflammatory damage in rats with TNBS-induced colitis, the underlying mechanisms of which may be attributed to its effects of promoting the expression of MMP-2 and MMP-9, leading to injury of the intestinal barrier.

## INTRODUCTION

The sulfur-containing amino acid homocysteine (Hcy) is a product of methionine demethylation metabolism and has various pharmacological effects, such as promoting oxidative damage, inducing the release of inflammatory mediators, stimulating the proliferation of vascular smooth muscle, and interfering with the lipid metabolism. With reference to cardiovascular diseases, Hcy has been proven to be involved in the pathological injury of atherosclerosis and thrombosis through inducing endothelial cell damage [1], up-regulating the expressions of chemokine and adhesion molecules [2], and affecting the normal function of immune cells [3]. Among these pathological changes, vascular endothelial cell injury has been recognized to be the key factor that contributes to the incidence and aggravation of cardiovascular diseases. Hcy may exert its effects of destroying the endothelial barrier, enhancing the permeability of endothelial cells, and driving inflammatory reaction by promoting the biosynthesis of matrix metalloproteinases (MMPs) [4, 5].

It has been reported that, in patients with inflammatory bowel disease (IBD), the Hcy levels in plasma and colonic mucosa are increased [6, 7]. Considering the structural and functional similarity between vascular endothelial cells and intestinal epithelial cells, we hypothesized that elevated Hcy levels in the plasma and colonic mucosa is involved in the pathophysiological process of IBD, by affecting intestinal permeability. We therefore developed a chemically induced chronic experimental model of mild hyperhomocysteinemia to investigate the influence of Hcy on the intestinal permeability in rats with TNBS/ethanol-induced colitis and to explore the potential mechanisms involved in this process. Through this study, we expect to contribute to the clarification of mechanisms of IBD development and provide experimental bases for improving intestinal permeability and promoting mucosal healing via decreasing the Hcy levels of patients with IBD.

## MATERIALS AND METHODS

### Animals and reagents

Male specific-pathogen-free (SPF) Sprague-Dawley rats, weighing 200 ± 20 g, were provided by Animal Center of Anhui Province. Animals were allowed one week of adaptation before entering the experimental period. Rats were housed at room temperature with a 12 h–12 h light–dark cycle. TNBS (031M5021), DL-Hcy (MW 135.18, 121M39044) and Evans Blue (MW 960.8) were purchased from Sigma (USA). Interleukin-1β (IL-1β), IL-6, tumor necrosis factor-α (TNF-α), myeloperoxidase (MPO) and malondialdehyde (MDA) test kits were obtained from R&D (USA). Matrix metalloproteinase-2,9 (MMP-2,9) test kits were purchased from Wuhan Xinqidi Biological Technology Co., Ltd. (China).

### Experimental assignment

The rats were divided into four experimental groups with eight in each group. Animals in Group A (normal control) were given normal saline enema and subcutaneous injection of normal saline. Rats in Group B (normal + Hcy injection) received normal saline enema and subcutaneous injection of Hcy. Group C rats (TNBS model) were given TNBS/ethanol enema and subcutaneous injection of normal saline. Group D rats (TNBS model + Hcy injection) received TNBS/ethanol enema and subcutaneous injection of Hcy.

### Establishment of model and drug application

According to the procedure reported previously [8], the animals were anesthetized by intraperitoneal injection of 10% chloral hydrate. Trinitrobenzene sulfonic acid (TNBS) dissolved in same volume of ethanol was administrated at the rate of 100 mg per kg *via* intracolonic insertion of a rubber tube, at a depth of 8 cm. Control animals received same volume of normal saline enema. DL-Hcy was dissolved in 0.9% normal saline and the pH value was adjusted to 7.4. According to the dosage previously reported [9], subcutaneous injection of Hcy (0.03 umol/g) was carried out twice per day with an 8-hour interval, from the first day after the establishment of TNBS/ethanol-induced colitis model. Hcy injection was continued at this rate for 30 days. Control animals received subcutaneous injection of normal saline.

### Sample collection

Animals were euthanized by intraperitoneal injection of 10% chloral hydrate. Blood samples were obtained from the abdominal aorta and centrifuged at 3000 rpm for 10 min. The supernatant was collected and stored at −80°C. The distal end of the colon and small intestine were both cut, with a length of 8 cm. The intestine was cut along the longitudinal axis and washed with ice-cold normal saline. Small bowel sacs were immediately prepared for determination of intestinal epithelial permeability. Parts of the colon were used for HE staining and colonic homogenate detection.

### Determination of Hcy levels in rat plasma and colonic mucosa

The Hcy levels in rat plasma and colonic mucosa were determined using high-pressure liquid chromatography-fluorescence detection (HPLC-FD) [10]. The plasma and colonic homogenate (100 uL) were mixed with 10 uL of tributylphosphine and incubated for 30 min at 4°C. Trichloroacetic acid (100 uL each) was added into the mixture and samples were incubated at room temperature for another 10 min. After centrifuging at 13 000 rpm for 10 min, 50 uL of the supernatant was collected into a new tube. The sample then was mixed with 10 uL of 1.55 mol/L NaOH, 125 uL of borate buffer solution and 50 uL of fluorescent reagent SBD-F and incubated in a 60°C water bath for 60 min. Twenty microlitres of the samples were used for HPLC analysis. The chromatographic conditions were as follows: samples were loaded onto an C 18 chromatographic column at room temperature. The mobile phase solution A was acetonitrile and solution B was phosphate buffer. The flow rate was set at 0.4 ml/min and the excitation and emission wavelengths were set at 385 nm and 515 nm, respectively.

### Evaluation of the severity of colitis

The rat body weight, as well as the stool consistency and frequency, was monitored daily during the experiment. Fecal occult blood test was performed daily to assess the fecal blood. The Disease Activity Index (DAI) was scored as previously described [11]. After experiments, rats were sacrificed under general anesthesia and 8 cm of the distal end colon was dissected, fixed with formaldehyde, embedded in paraffin, and stained with HE to evaluate histological index (HI) scoring as also previously described [12].

### Colonic homogenate detection

Colonic tissues were prepared as 10% colonic tissue homogenate. According to the manufacturer’s instructions, the MPO level was examined by the tetramethyl benzidine method and the MDA level was examined by thiobarbituric acid method. IL-1β, IL-6, TNF-α, MMP-2, and MMP-9 levels were evaluated by ELISA.

### Examination of the intestinal epithelial permeability

The intestinal epithelial permeability was examined by Evans Blue (EB) assay [13]. A small bowel sac of 8 cm length was prepared and infused with 0.3 mL EB solution. After washing with normal saline, the small bowel sac was dried for 24 h, weighted, and incubated with 1 mL of formamide for 24 h. After centrifuging, the supernatant was collected and examined under an ultraviolet spectrophotometer at a wavelength of 620 nm. The EB content in tissues was calculated according to a standard curve.

### Statistical analysis

Data were analysed using SPSS 13.0 software (IBM Corp.). Data were presented as means ± standard deviation (SD). Statistical significance between different groups was determined using one-way analysis of variance (ANOVA). Least significant difference (LSD) *t-*test was used for multiple comparisons within the same group.

## RESULTS

### Model establishment and drug administration outcomes

**Figure 1.**
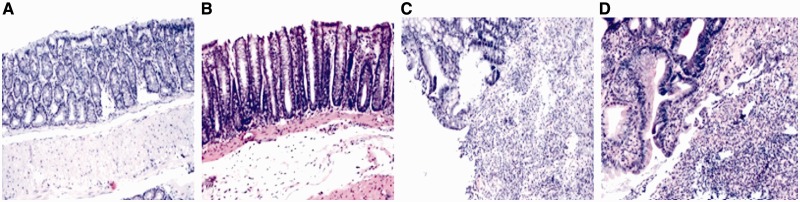
Histopathological features of the colon in association with colitis (HE × 40).

**Figure 2.**
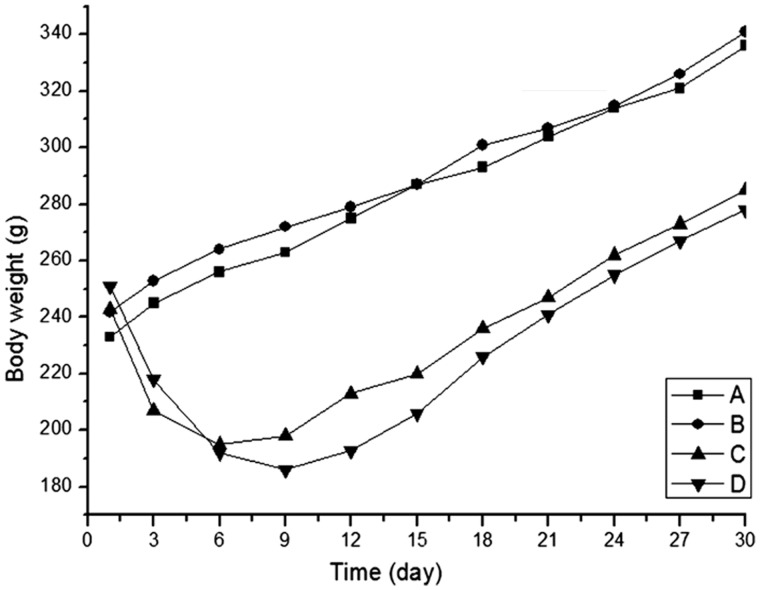
Body weight changes of rats with TNBS-induced colitis during 30 days (*n* = 8).

**Figure 3.**
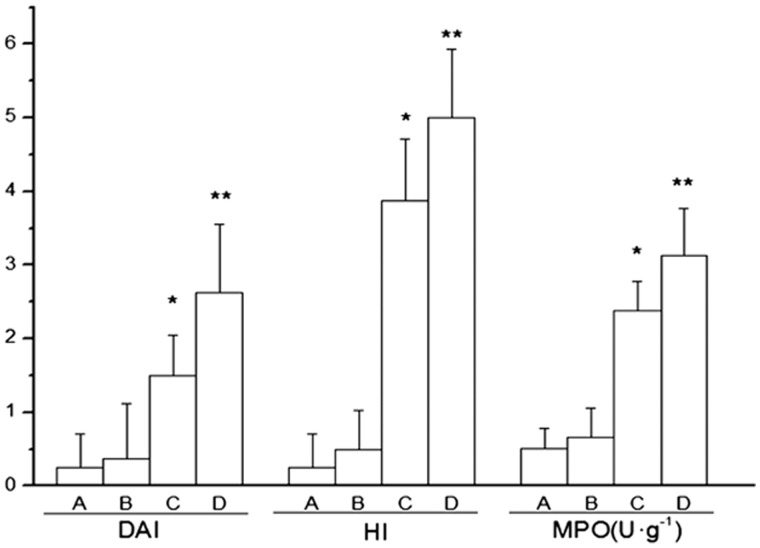
Effect of Hcy on DAI scoring, HI scoring, and MPO activity in rats with TNBS-induced colitis (*n* = 8). *P < 0.01 *vs* Group A; **P < 0.01 *vs* Group C.

**Figure 4.**
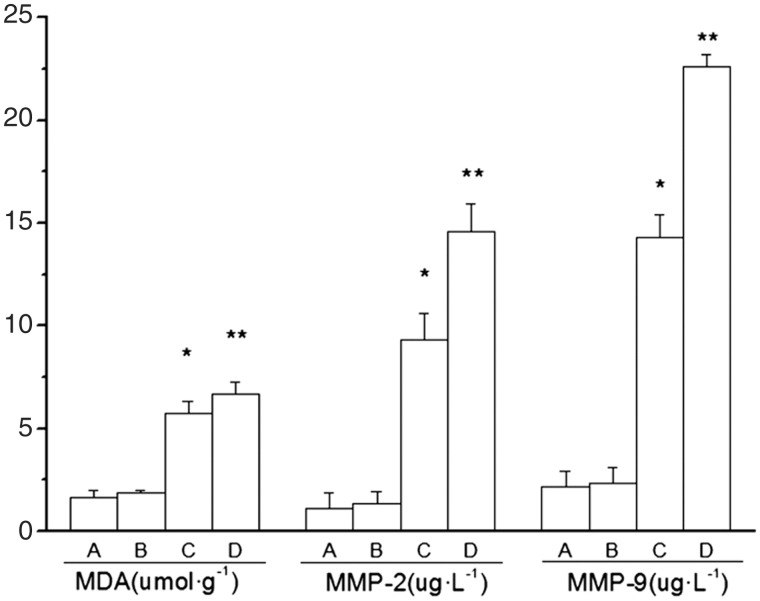
Effect of Hcy on the content of MDA, MMP-2, and MMP-9 in rats with TNBS-induced colitis (*n = 8*). *P < 0.01 *vs* Group A; **P < 0.01 *vs* Group C.

All animals survived until the end of experiment. Decreased food intake and body weight, accompanied with a yellow-haired and dispirited appearance, were observed within two days of the establishment of the colitis model. Digestive tract symptoms such as loose stool and fecal blood were also observed. Histological examination showed that mucosa and submucosa were the main sites of colonic damage, with abundant inflammatory cell infiltration, local erosion and ulcer formation (Fig. 1). Compared with those in Group A, animals in Groups C and D exhibited obviously reduced body weight (Fig. 2) and increased DAI and HI scores (Fig. 3). The MPO activity and MDA content were up-regulated in colonic homogenate (all *P* < 0.01) (Figs. 3 and 4), indicating the successfully established rat colitis model. After 30 consecutive days of subcutaneous injection, the Hcy levels in the plasma and colonic mucosa of Groups B and D were significantly increased, as compared with Group A (*P* < 0.01; Table 1).

**Table 1.** Hcy levels in plasma and colon mucosa after chronic administration and its effect on the content of EB in rats with TNBS-induced colitis (*n* = 8)

Group	EB (ug/g)	Plasma Hcy (umol/L)	Colon mucosa Hcy (umol/g)
A	106.32 ± 7.26	4.09 ± 0.91	1.12 ± 0.27
B	116.50 ± 17.12	19.04 ± 1.65^a^	2.64 ± 0.43^a^
C	610.21 ± 11.56^a^	11.46 ± 1.93^a^	2.16 ± 0.31^a^
D	829.95 ± 11.42^ab^	24.57 ± 2.18^ab^	3.29 ± 0.47^ab^

^a^*P* < 0.01 *vs* Group A; ^b^*P* < 0.01 *vs* Group C

### Influence of Hcy on the colonic injury and intestinal permeability of TNBS-induced colitis

As compared with Group C, the DAI, HI scores, MPO activity, and MDA content were significantly increased in Group D (*P* < 0.01) (Figs. 3 and 4), suggesting that Hcy may aggravate oxidative and inflammatory injury in rats with TNBS-induced colitis. In addition, the EB content of the intestinal mucosa of Group D was significantly higher than that of Group C (*P* < 0.01; Table 1), suggesting that Hcy may increase the intestinal permeability of rats with TNBS-induced colitis.

### Influence of Hcy on the inflammatory factors and MMP levels of TNBS-induced colitis

**Figure 5.**
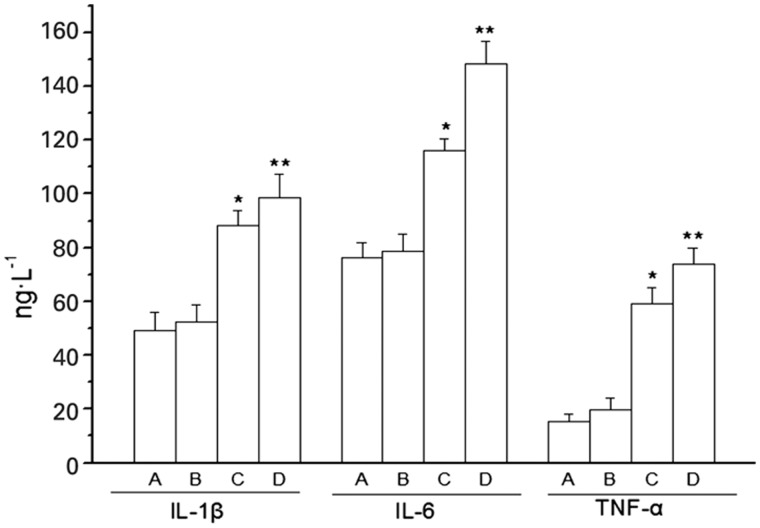
Effect of Hcy on the levels of IL-1β, IL-6, and TNF-α in rats with TNBS-induced colitis (*n = 8*). *P < 0.01 *vs* Group A; **P < 0.01 *vs* Group C.

Compared with those of the control group, the levels of IL-1β, IL-6, TNF-α, and the contents of MMP-2 and MMP-9 in the colonic tissues were significantly increased in Group C and -D rats. Also, Group D rats showed more obvious elevation of the above index values, as compared with Group C rats (*P* < 0.01; Figs. 4 and 5), implying that Hcy may aggravate colonic damage by triggering the release of inflammatory factors in rats with colitis, and may impair the intestinal barrier by increasing expression of MMPs.

## DISCUSSION

TNBS is a hapten, but will turn into an antigen when it is bound with macromolecular substances. TNBS can penetrate into the colon and induce colitis after ethanol-induced injury of the intestinal barrier, which is similar to human Crohn's disease (CD) in its pathological features [14]. In this study, the body weights of rats with colitis were significantly decreased and exhibited different degrees of fecal blood with increased DAI and HI scoring and elevated MPO activity, suggesting that a rat colitis model had been successfully established, induced by TNBS/ethanol.

Hcy can induce the generation of reactive oxygen (ROS) and elicit oxidative damage by various means, including activating the NADPH oxidase and p38 MAPK signal pathways. Hcy can up-regulate the expressions of many pro-inflammatory factors, such as TNF-α, IL-6, and IFN-γ, and plays a critical role in the denaturation of key molecules through the hypomethylation of DNA and protein. Hence, Hcy is considered to be one of the risk factors for cardiovascular- and end-stage renal diseases [15–18]. The association between Hcy and IBD has been extensively studied. It has been demonstrated that the elevation of Hcy levels in the plasma and colonic mucosa of patients with IBD might be related to the decreased levels of plasma folic acid and Vitamin B_12_ (VitB_12_), as well as the gene polymorphism of Hcy metabolic enzymes [10, 19]. Chen *et al.* reported that plasma Hcy level was obviously increased in rats with a restricted intake of VitB_12_ and folic acid [20]. Here, our results showed that the Hcy levels in plasma and colonic tissues of rats with colitis were increased significantly, which might be associated with the long-term decreased food intake and the loss of vitamin supplements due to severe diarrhea. In this study, the DAI and HI scores, MPO activity in colonic tissues, as well as the MDA, TNF-α, IL-1β, and IL-6 levels, were simultaneously increased in TNBS-induced colitis rats with Hcy injection, indicating that Hcy can aggravate the oxidative and inflammatory damage in rats with colitis.

An intact intestinal barrier contributes to maintaining stability of the intestinal flora, inhibiting the migration of intestinal bacteria and toxins, and avoiding damage induced by food antigens and microorganisms. Therefore, damage of the intestinal barrier may aggravate mucosal inflammation and immune responses and is believed to be the key process of infection- and immune factors-initiated intestinal infection. The mechanical barrier, which is comprised of intestinal mucosal epithelial cells and cell–cell tight junctions, is the structural basis of the intestinal barrier. Ye *et al.* showed that TNF-α could up-regulate the expression of myosin light chain kinase, which played a key role in regulating intestinal permeability, destroying cell junctions, and increasing cell permeability in Caco-2 cells [21]. Capaldo *et al.* indicated that pro-inflammatory factors, such as TNF-α, IL-1β, IL-6, and IFN-γ, enhanced intestinal mucosal permeability by destroying the cell–cell tight junctions [22]. Rao *et al.* demonstrated that restricting the intake of methionine could improve intestinal barrier function and promote the recovery of damaged mucosa [23]. In the present study, Hcy injection of rats with colitis simultaneously resulted in increased EB content and TNF-α, IL-1β, IL-6 levels as compared with the colitis control group, suggesting that Hcy can promote the release of pro-inflammatory factors in the colonic tissues of rats with colitis, increase intestinal mucosal permeability, and further aggravate intestinal inflammation.

As important proteolytic enzymes in the human body, MMPs play an essential role in regulating the metabolism of the extracellular matrix (ECM). Oxygen free radicals and inflammatory factors can promote the expression of MMPs subsequently destroying the intact intestinal mucosal structure and increasing mucosal permeability by degrading cell–cell tight junctions, basal membrane, and ECM [24]. Hcy has been proven to promote the activity and increase the expression of MMPs. Bescond *et al.* found that low concentrations of Hcy enhanced the activity of MMP-2 [25]. Mice with deficiency in the cystathionine β synthase (CBS) gene may develop high levels of Hcy in plasma. Kumar *et al.* showed that the levels of MMP-2 and MMP-9 were up-regulated in the brain tissues of the CBS knockout mice [26, 27]. In this study, the colitis rats with Hcy injection simultaneously showed increased EB content and MMP-2, MMP-9 levels as compared with the colitis control group, suggesting that Hcy can increase intestinal mucosal permeability, the underlying mechanisms of which may be attributed to its effects of promoting inflammatory injury and expression of MMP-2 and MMP-9, leading to injury of the intestinal barrier.

In summary, using TNBS/ethanol enema and subcutaneous injection of Hcy in rats, we found that Hcy significantly aggravated intestinal inflammatory injury and increased intestinal permeability in rats with colitis. We speculate that th Hcy-mediated pro-inflammatory responses and elevation of expression of MMPs might be involved in this process. However, the mechanism of Hcy-induced intestinal barrier damage has not yet been well illustrated. Future study will continue to examine expression of the tight-junction-associated molecules, such as occludin, claudin and ZO-1, in intestinal epithelial cells. Electron microscopy will also be conducted to understand the alternations of subcellular structures.

**Funding:** This study is supported by The Natural Science Foundation of Anhui Province (1308085MH146), and Fund of Yang Sen Science Research Council China (2012JRCC Digest 02).

**Conflict of interest:** none declared.
